# Protection against Cancer with Medicinal Herbs via Activation of Tumor Suppressor

**DOI:** 10.1155/2012/236530

**Published:** 2012-11-20

**Authors:** Yasuko Kitagishi, Mayumi Kobayashi, Satoru Matsuda

**Affiliations:** Department of Environmental Health Science, Nara Women's University, Kita-Uoya Nishimachi, Nara 630-8506, Japan

## Abstract

Cancer remains a major cause of death, although research is ongoing for the development of more effective drugs. Some herbs have shown potential in preventing the occurrence and/or progression of cancer and other chronic diseases. They are being screened comprehensively to explore the possibility of development of feasible anticancer drugs. However, more information is required about the response to and the molecular target for specific herbs. It seems that there is a relationship between some medicinal herbs and tumor suppressor molecules which protect a cell from cancer. In this paper, we summarize the progress of recent research on herbs, with a particular focus on its anticancer role and molecular mechanisms underlying the cancer prevention property, supporting design for further research in this field.

## 1. Introduction

Many herbs have been used for a long time for demanded health benefits [1–3]. The medicinal herb is a plant (or a plant part) used for therapeutic properties. It is a widely recognized fact that many pharmacologically active drugs are derived from natural resources such as medicinal plants [4, 5]. Therefore, it is reasonable to search for novel drug molecules in herbs. Actually, herb-derived compounds have provided attractive possibilities for treatment strategies. Herbal medicine products are also provided as dietary supplements that people take to improve their health. For example, St. John's wort has been used in the treatment of anxiety, stomach upset, insomnia, and so on. Historically, herbs have enjoyed a rich tradition of use both for their flavor enhancement and for their medicinal properties. While herbs present interesting possibilities for health promotion, more information is required about the physiological and pharmacological responses to and the molecular targets for specific herbs. 

This paper focuses on antitumorigenic properties and anticancer contributions of herbs. Epidemiological evidences also point to herbs as dietary constituents with multiple anticancer characteristics [6]. It would be important to define appropriate strategies to achieve benefits from medicinal herbs. Studies show that some herbs can inhibit some antiapoptotic genes and activate some apoptotic genes [7, 8]. Systematic characterization of active components in medicinal herbs and their mechanisms of action is important for providing the rationale for their efficacy. Therefore, biotechnological work has to be done in order to provide evidence for the efficacy and to bring herbs and derived compounds to clinical use. In this paper, the relationship between medicinal herbs and some tumor suppressor molecules has been reviewed with a focus on gene expression and posttranslational modifications.

## 2. Herbal Medicine in Cancer Prevention

Herbal medicine has been used since early times to treat malignancies in Asian countries [9]. The association between dietary patterns and the risk of developing breast cancer has also been shown in an Italian cohort, suggesting that a diet rich in raw vegetables and olive oil protects against breast cancer [10]. Furthermore, individuals consuming more raw vegetables, herbs, and spices have been associated with lower cancer risk [10]. Natural products are of importance in devising new drugs and providing unique ideas in cancer therapy. Actually, some herbs or spices have been approved to have a character of anticancer [11]. For example, the consumption of curcumin, a component of curry turmeric powder, has been reported to be a factor linked to a lower incidence of colon cancer [12]. Cells resistant to certain apoptotic inducers and/or radiation become susceptible to apoptosis when treated with curcumin. In addition, curcumin can also act as a chemopreventive agent in several cancers by suppressing colonic aberrant crypt foci formation and DNA adduct formation [12]. Furthermore, many cytotoxic chemotherapeutic agents such as etoposide are originally purified from herbs [13]. 

Two mechanisms have been proposed to be responsible for the anticancer action of the herbs and spices. One is via direct cytotoxic effects and the other is indirect through immunological modulatory action. The occurrence of cancer cells may be caused by the abnormal proliferation of cell or the inhibition of cellular apoptosis pathway. Many kinds of genes are involved in the cell proliferation and/or apoptotic regulation of cancer cells. The proliferation and the apoptosis of tumor cells are also affected by many factors and pathways such as drugs, radioactive ray, medicinal herbs, via the modulation of some oncogenes or tumor-suppressor genes. One of the potential anticarcinogenic mechanisms of herbs is via an immunological modulation. It is supposed that anticancer action of medicinal herbs may be attributed to its power to exercise immune potentiation, which may have various therapeutic applications in prophylaxis of opportunistic infections and malignant diseases. In recent years, an active ingredient responsible for the immunomodulation of some herbs has been found to be a form of complex polysaccharides [14]. As several plants have potential medical and biological efficacy used by patients with oncological neoplasia, further studies are necessary to evaluate those efficacies, where the efficacy is based on the molecular mechanisms.

## 3. Activation and Inactivation of Tumor ****Suppressor Molecules

**Figure 1:**
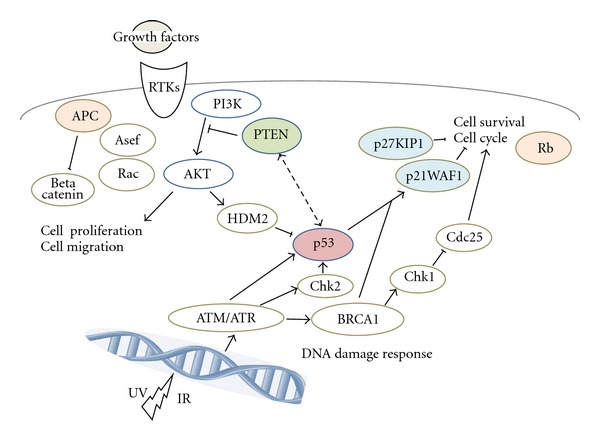
Schematic representation of tumor suppressor signaling including p53, Rb, APC, p21WAF1/p27KIP1, and PTEN. Examples of molecules known to act on cell proliferation and cell survival via the regulatory pathways are shown. Note that some critical pathways have been omitted for clarity.

**Figure 2:**
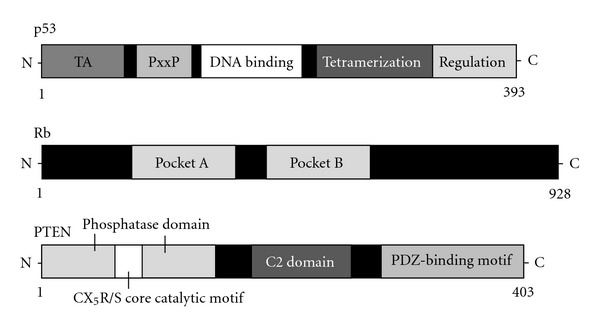
Schematic structures of p53, Rb, and PTEN proteins. The predicted consensual domain structures for each protein are depicted. The functionally important sites are also shown. Note that the sizes of protein are modified for clarity. TA: transactivation domain; PxxP: proline-rich region; pocket A, pocket B: a tandem of folds; C2 domain: a protein structural domain involved in targeting proteins to cell membranes; PDZ: a common structural domain in signaling proteins (PSD95, Dlg, ZO-1, etc.).

Tumor development may be accelerated by disruption of the balance between cell proliferation and cell death, which is maintained through regulations of various signal transduction pathways. Apoptosis, also known as programmed cell death, is caused by various cell injuries including DNA damage [15]. It has been demonstrated that various cell proliferation- and apoptosis-signal transduction pathways are built on complicated networks between oncogenes and tumor suppressor genes such as p53 and its downstream factors [16]. For example, a tumor suppressor p53 controls various genetic expressions and plays an important role in cell proliferation and in modulation of signal transduction pathways. Accumulation of p53 in cells after DNA damage leads to cell cycle arrest and apoptosis induction. In addition, p53 is involved in repair of damaged DNA and, thus, prevents accumulation of mutations and suppresses tumor development [17]. Tumor suppressor molecules protect a cell from cancer. When the tumor suppressor genes lose their function, the cell may progress to cancer in combination with other genetic changes. Tumor suppressor genes regulate diverse cellular activities including DNA damage repair, cell proliferation, cell differentiation, cell migration, and programmed cell death (Figure 1). An important tumor suppressor is the p53 tumor suppressor. Other examples of tumor suppressors include Rb, PTEN, p21WAF1, p27KIP1, and APC (Figure 2) [18]. The recessive characteristics of these genes require mutations on both alleles. The p53 gene product is a transcriptional factor that blocks the progression of cell cycle. Mutations in the p53 gene are frequently found in many human cancers, and the mutation sites are localized in the conserved region of the gene [19]. Consequently, it loses its ability to bind its consensus DNA sequences, and it can neither activate the target genes nor perform its tumor-defensive functions during carcinogenesis. There are two types of p53 genes: the wild-type p53 gene and the mutant p53 gene [20]. Cancerous p53 mutations usually confer the mutant protein with a dominant-negative activity over the remaining wild-type gene. Moreover, many mutant p53 forms acquire dominant-negative activities and sometimes gain oncogenic properties [21]. These activities of p53 are also regulated by posttranslational modification. Phosphorylation and acetylation state, subcellular localization, and interaction with other cellular proteins are likely to influence the function of p53 [22]. In cells facing oxidative stress and DNA-damage, for example, p53 dissociates from its ubiquitin ligase MDM2 [23], via various posttranslational modifications which promote its stabilization and activation.

Rb gene alteration or functional inactivation of pRb, an Rb gene product, has also been reported in several kinds of cancers. The phosphorylation of the pRb is regulated by cyclin-dependent kinases in accordance with the cell cycle [24]. Hypophosphorylated wild-type pRb is tightly bound to the nuclear matrix and seems to be critical in the inhibition of cellular proliferation. Hyperphosphorylation is a physiological mechanism of inactivation of pRb [25]. Many cell cycle regulators modulate pRb function through its phosphorylation status. Active complexes of G1 cyclins and cyclin-dependent kinases inactivate pRb through its phosphorylation, while p21WAF1 and p27KIP1 inhibit cyclin-dependent kinases [26]. The pRb protein appears to prevent the function of transcription factors and other proteins needed for S phase until its inactivation by cyclin-dependent kinases in late G1. Deregulated inactivation of pRb in G1 phase may be a universal mechanism underlying cellular transformation. As reversible protein phosphorylation plays a central role in regulating intracellular signaling, dysregulation of the mechanisms that regulate phosphorylation may play a key role in cancer initiation and maintenance. Works had focused on the role of specific protein phosphatases in malignant transformation [27].

The phenotype of the cancerous cell may arise from epigenetic events that may alter the heritable state of gene expression. Epigenetic silencing of the tumor suppressor genes is a well-established oncogenic process [28], and the reactivation of tumor suppressor genes is an attractive molecular target for cancer therapy. Epigenetic alterations, such as DNA methylation, also appear to be tightly linked to the sequential nonreversible events of normal tissue differentiation and organogenesis [29]. Hypermethylation of CpG islands, an epigenetic event that is not accompanied by changes in DNA sequence, represents an alternative mechanism to inactivate tumor suppressor genes and plays a major causal role in cancer [30]. As a result of histone deacetylation and methylation, the CpG island's hypermethylation establishes a state of gene silencing. Accumulating evidence indicates that CpG island's hypermethylation is an early event in cancer development [31]. 

## 4. Relationship between Medicinal Herbs and Tumor Suppressors

**Figure 3:**
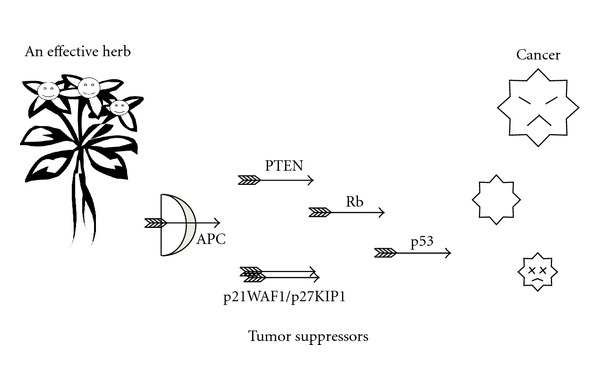
Tumor-suppressor-dependent anticancer function of a medicinal herb. Schematic illustrations of the tentative model for the anticancer function of herbs are shown. Herbs can stimulate tumor suppressor activities against cancer, which can also contribute to cancer prevention.

Many herbs had been established to have a character of antitumor activity (Figure 3). Experimental studies about antitumor effects of herbs had also been found [32]. On the other hand, many kinds of tumor suppressor genes had been shown to be involved with cell proliferation and apoptotic regulation of cancer cells. There might be a substantial relationship between medicinal herbs and tumor suppressors.

### 4.1. p53

*Scutellaria baicalensis* is used as an adjuvant to cancer chemotherapy. Following the treatment, the altered cellular protein expression indicates that cell growth arrest and apoptosis are potential mechanisms of its cytotoxicity. Increased expression of p53 in the cancer cells of key proteins related to the enhancement of apoptosis is observed [33]. Similarly, *Gleditsia sinensis *thorns are used as a medicinal herb, which shows a decrease in cell growth and an increase in cell cycle arrest during the G2/M-phase. The arrest is correlated with increased p53 levels and downregulation of cyclinB1 [34]. The mRNA level of the wild-type p53 gene also increases with the treatment of Kanglaite, an extract from Coix seed. It can be assumed that Kanglaite extends half-life of p53 protein, by which Kanglaite may induce the apoptosis of tumor cell [35]. A Ginsenoside, one of components in American ginseng herb, increases levels of Bax protein and induces cell death, activating the p53 tumor suppressor [36]. Knockout of p53 dramatically decreases the cell death, suggesting that p53 contributes to apoptosis induced by Ginsenoside in the cancer cells. Thymoquinone, the most abundant component in black seed, is a dietary chemopreventive agent against cancer. Apoptosis induction by thymoquinone is associated with an increased p53 mRNA expression and the downstream p53 target genes [37]. Treatment with a specific inhibitor of p53 restores p21WAF1 level to the untreated control and suppresses the cell cycle arrest and apoptosis. The apoptotic effect of thymoquinone is, thus, linked to p53.

### 4.2. Rb

The honokiol, a component of oriental herb *Magnolia officinalis*, treatment decreases the viability of PC-3 and LNCaP human prostate cancer cells in a concentration- and time-dependent manner with G0-G1 phase cell cycle arrest. The honokiol-treated PC-3 and LNCaP cells exhibited a marked decrease in the levels of total retinoblastoma protein (Rb), which correlated with the suppression of transcriptional activity of E2F1 [38]. Triptolide, a purified extract from a herb *Tripterygium wilfordii hook F*, exhibits antiproliferative and proapoptotic function. Triptolide shows increased p21(cip1) expression and reduced pRb phosphorylation [39]. *Acanthopanax gracilistylus*, a medicinal herb, markedly inhibits the proliferation of several cancer cell lines such as MT-2, Raji, HL-60, TMK-1, and HSC-2 [40]. The mechanism of the inhibition of the cell growth involves arrest of the cell cycle at the G(0)/G(1) stage, being accompanied by a decrease of phosphorylated pRb protein and reduced Cdk2 and Cdk4. Licochalcone is a novel estrogenic flavonoid isolated from a herb licorice root and shows antitumor activity in various human cell lines. Licochalcone also inhibits phosphorylation of pRb, specifically phosphorylation of S780, and reduces expression of transcription factor E2F; cyclins D1, Cdk4, and Cdk6 [41, 42]. Similarly, Dichloromethane from cape aloe extract inhibits cell proliferation, which is also associated with decreased pRb phosphorylation [43]. In addition, multiherb anti-inflammatory product Zyflamend downregulates phosphorylation of pRb protein [44]. 

### 4.3. PTEN

The Honokiol is demonstrated to attenuate the angiogenic activities of human endothelial cells, which can attenuate the PI3 K/Akt/mTOR (mammalian target of rapamycin) signaling by downregulation of Akt phosphorylation and upregulation of PTEN (phosphatase and Tensin homolog deleted on chromosome 10) expression [45, 46]. Combination of honokiol with the mTOR inhibitor rapamycin presents synergistic effects on induction of apoptosis of cancer cells. Curcumin, an active ingredient derived from the rhizome of the plant *Curcuma longa*, has anticancer activity. PTEN enhances curcumin-induced apoptosis, whereas inactive PTEN (G129E and G129R) inhibited curcumin-induced apoptosis [47]. *In vitro* studies have revealed that curcumin and resveratrol synergistically inhibit cell growth and induce apoptosis [48]. Molecular targets including phosphorylated Akt, cyclinD1, mTOR, and androgen receptor are down-regulated by the combination of curcumin and resveratrol due to the activation of PTEN, suggesting that some herbs may reduce cancer incidence due to the PTEN function. However, some component(s) of Rosemary herb inhibits the expression of PTEN in K562 myeloid cell line cells [49].

### 4.4. p21WAF1 and p27KIP1

An extract of *Magnolia officinalis* inhibits cell proliferation in cultured urinary cancer cells. The inhibition of proliferation is associated with G1 cell cycle arrest. Treatment with *M. officinalis* extract upregulates the expressions of p21WAF1 and p27KIP1, which are CDK inhibitors [50]. The protein product of p21WAFI can combine with cell circle protein, Cdks, and proliferating cell nuclear antigen to form a complex that can depress the cell growth. *M. officinalis*-extract-induced cell growth inhibition also appears to be linked to the activation of p38 MAP kinase through the p21WAF1 expression [50]. Baicalin, a herb-derived flavonoid compound, has been shown to induce apoptosis and growth inhibition in cancer cells through multiple pathways. Baicalin may be a novel, adjunctive therapy for malignancies including prostate cancer. Baicalin inhibits the proliferation of LNCaP and PC3 prostate cancer cell lines. Concomitantly, baicalin enhances the expression of the cyclin-dependent kinase inhibitor, p27 Kip1, in the LNCaP cells [51]. Treatment with ethanol extract of *Gleditsia sinensis *thorns on vascular smooth muscle cells leads to a decrease in cell growth by arresting cells in the G2/M-phase of the cell cycle, which is associated with upregulated p21WAF1 levels [52]. Conversely, the p21WAF1 expression is blocked by treatment with the p38 MAPK-specific inhibitor SB203580. While upregulating the expression of p53 protein, Kanglaite can also raise the expression of p21WAF1, suggesting that Kanglaite can induce apoptosis of cancer cell by way of the p53-dependent manner. However, treatment with ethyl acetate extract of *Saussurea involucratat* results in growth inhibition with G1 phase cell cycle arrest and apoptosis in PC3 cells. The treatment also induces p21WAF1 and p27KIP1 expression, independent of the p53 pathway [53].

### 4.5. APC

The mRNA and protein expression level of the tumor suppressor genes adenomatous polyposis coli (APC) is increased following treatment with trichosanthin, a bioactive component isolated from herbal plant *Trichosanthes kirilowii *Maximowicz, in HeLa cells [54]. Methylation-specific PCR (MSP) detection showed that trichosanthin induced demethylation in the HeLa cells and that this de-methylation activity was accompanied by the decreased expression of DNMT1 and reduced DNMT1 enzyme activity. Trichosanthin is capable of restoring the expression of methylation-silenced tumor suppressor genes. It is potentially useful as a de-methylation agent for the clinical treatment of cancers. Carnosol, a component of rosemary, prevents APC-associated carcinogenesis, potentially via its ability to enhance E-cadherin-mediated adhesion and suppress beta-catenin tyrosine phosphorylation [55]. Dietary administration of carnosol may decrease intestinal cancer cell growth.

### 4.6. Perspective

In conclusion, medicinal herbs could serve as a promising approach for cancer treatment. The information here may provide further insight into the molecular mechanisms underlying the clinical use of the herbs (e.g., ginsenoside and Kanglaite) as a cancer therapy. The identification of target molecules relevant to diseases allows screening for natural products capable of modulating these targets. This may represent the basis for the development of rational treatment of diseases such as cancer. As it is important to identify a set of genes related to the drug's sensitivity to cancer cells in order to establish a predictive method, this kind of research also opens avenues for the prediction of the response of individual cancer patients to therapy. As we have already had knowledge and experience on medicinal herbs for centuries, drug developments from the natural herb products should be differently approached from the conventional pharmaceutical pattern. Indeed, the isolation and elucidation of their chemical structure enable pharmacological and molecular biological investigations comparable to chemically synthesized compounds. It is, however, not possible to predict which individual cases of cancer patients will respond to proposed therapy. The concept of individualized tumor therapy is of importance for traditional herbal medicines. Therefore, detailed mechanisms underlying the effects of herbs should be further investigated in the near future, aiming to obtain such information for the potential of increasing cancer specificity and reducing adverse side effects on normal tissues.
